# Gaining consensus on emotional wellbeing themes and preferences for digital intervention type and content to support the mental health of young people with long‐term health conditions: A Delphi study

**DOI:** 10.1111/hex.14025

**Published:** 2024-04-09

**Authors:** Jennie Brown, Lauren Cox, Kathleen Mulligan, Stephanie Wilson, Michelle Heys, Polly Livermore, Suzy Gray, Angeliki Bogosian

**Affiliations:** ^1^ School of Health and Psychological Sciences City University of London London UK; ^2^ School of Mathematics, Computer Science and Engineering City University of London London UK; ^3^ East London NHS Foundation Trust London UK; ^4^ Population, Policy and Practice Department University College London London UK; ^5^ Great Ormond Street Institute of Child Health (GOS ICH) London UK

**Keywords:** Delphi method, digital interventions, mental wellbeing, young people with long‐term conditions

## Abstract

**Background:**

Young people (YP) with long‐term conditions (LTCs) are at greater risk of psychological distress than those without LTCs. Despite this, there is a scarcity of quality digital interventions designed to help improve mental wellbeing in this population. The aim of this study was to determine what YP, parents and health professionals preferred for future interventions.

**Methods:**

Twenty‐six YP with asthma, diabetes and/or epilepsy (the three most common LTCs in YP), 23 parents of YP with LTCs and 10 health professionals mainly in paediatric specialisms (total *n* = 59) took part in an online Delphi study to gain consensus (set at 75% agreement) on four questions across three rounds. Participants ordered psychological themes that may be experienced by YP with LTCs by importance and ranked digital intervention types and delivery modes by importance or usefulness. The most common results were reported if no consensus was reached by round 3.

**Results:**

Participants preferred a mobile phone app (73% agreement) and a mixture of one‐on‐one and group support for an intervention (75% agreement). The two highest ranked psychological themes were anxiety (44%) and wanting to appear ‘normal’ (38%), and the top intervention type was ‘general counselling’ (54% agreement).

**Conclusion:**

There was a clear desire for an app to help with the psychological aspects of living with LTCs and for a combination of one‐to‐one and group intervention elements. Anxiety and wanting to appear ‘normal’ might be two closely linked psychological challenges that could be addressed by a single intervention.

**Implications:**

The results will be important to consider for a future intervention, although further consultation will be needed for app development.

**Patient or Public Contribution:**

Two YP with a LTC provided feedback on the study protocol including the aims and procedures of the project. Another six YP with LTCs were consulted on an early draft of the study questionnaire (the four questions), which was subsequently revised. Once the project began, a patient and public involvement group consisting of two YP with LTCs and one parent of a YP with an LTC gave feedback on the research process, lay report of the results and dissemination plan.

## 1 INTRODUCTION

One in 10 children will develop a long‐term condition (LTC) that limits their daily life substantially and demands extended care, supervision and self‐management strategies.^1^ Children and adolescents with LTCs have significantly higher rates of mental health problems compared to ‘healthy’ children.^2^, ^3^ The World Health Organization (WHO) has therefore called for more research into potential interventions to support young people with a LTC.^3^

In this paper, we will focus on the three most common LTCs in children in the United Kingdom: asthma, diabetes and epilepsy.^4^ Approximately one in 11 children have asthma,^5^ 35,000 under 19‐year‐olds have diabetes^6^ and around one in 220 children have epilepsy.^7^ These LTCs typically require constant monitoring, adherence to treatment plans and often lifestyle modifications for symptom management and avoiding complications, such as lung infections in asthma and hypoglycaemia (low blood glucose) in type 1 diabetes.

As a result of their complex management, these conditions heavily affect the lives of young people living with them. There is a significantly higher occurrence of anxiety and depression symptoms in young people with epilepsy than those without epilepsy.^8^, ^9^ The findings of heightened anxiety and depression are echoed in meta‐analyses looking at both diabetes^10^, ^11^ and asthma.^12^ Meta‐analyses also showed that young people with asthma are three times more likely to experience anxiety compared to those without asthma^13^ and a third of young people with diabetes experience distress that consequently leads to poor blood sugar control, decreased self‐care and low self‐efficacy in condition management.^14^

Adolescence is already a difficult time for most, yet the stress of managing an LTC likely adds to this challenging period and impacts the management of the condition and, consequently, physical health. According to a recent longitudinal study, children with LTCs were 50% more likely to have mental health issues at age 10 and 13, and 60% more likely at age 15, compared with children without LTCs.^15^ In particular, there is a strong relationship between asthma and diabetes in adolescence and an increase in the prevalence of anxiety and depressive disorders.^16^, ^17^ Further, several associations between mental health and subsequent physical conditions start being significant in LTCs in childhood/adolescence.^18^ Adolescence is not only critical due to these associations with increased risk of mental health and additional physical conditions but also as a key period where many adolescents with LTCs are beginning to take charge of managing the condition themselves, as they gain more independence. There is a need to help young people prepare for the responsibility of managing their health.^19^, ^20^

Psychological therapies can support those with LTCs.^21^, ^22^ Cognitive behavioural therapy (CBT) is effective in helping young people with epilepsy manage their psychological symptoms such as anxiety and low mood.^23^ Group therapy helped young people with epilepsy to increase their knowledge of epilepsy and discuss epilepsy with others^24^ and improved diabetes‐related quality of life in young people with diabetes.^25^ Psychological interventions have also been linked with improvements in physical health markers such as reduced glycated haemoglobin levels in young people.^26^

Providing psychological support using technology is well received within the young population.^27^ A recent scoping review showed that technologies such as mobile apps and websites can assist the self‐management of LTCs, are an acceptable method of delivering information and can promote the development of effective self‐management skills by parents and children.^28^ However, the authors have also identified the need for future technology design to include children and parents in all stages of development.

The use of digital interventions (activities provided via technologies such as computer, smartphone, virtual reality, etc.) for improving mental wellbeing in YP with LTCs is relatively recent. A meta‐analysis^29^ and two systematic reviews^30^, ^31^ list a relatively small number of digital interventions for this population, which were either internet‐based, mobile phone‐based or a combination of both. The predominant underlying therapeutic technique for these interventions was CBT. Other interventions were based on a wide range of other techniques or theories, for example, dialectic behavioural therapy, problem solving, social cognitive theory, acceptance and commitment therapy, relaxation and mindfulness or a combination of different approaches.

The efficacy of such digital interventions is a young research field with a relatively small number of randomised controlled trials (RCTs).^29^, ^30^, ^31^ A meta‐analysis of 19 studies assessing Internet‐ and mobile‐based interventions (mainly CBT based) for YP with LTCs reported improvements in self‐efficacy and disease‐related measures but no significant differences in symptoms of depression and quality of life.^29^ The authors suggest finding ways to improve the potential efficacy of interventions by including young people's views during intervention development and looking at possible moderators of treatment outcomes such as parental support.

Ensuring interventions are aligned with service users' and stakeholders' priorities can increase the relevance, acceptability and ultimately efficacy of these interventions.^32^ The results of this study will show us which psychological theme to focus on and what modes of intervention delivery are preferred before embarking on a more detailed intervention co‐design process.

This study aimed to prioritise the focus, content and tools that could be used in a digital intervention to support the mental health of young people with LTCs. Our objectives were:

1. (FOCUS): To identify and prioritise key mental health themes that young people may experience.
2. (CONTENT): To identify preferences for psychological techniques and interventions to be incorporated.
3. (TOOLS): To identify preferences for digital tools (e.g., website, mobile app, social media, virtual reality) that could be used in a future mental health intervention.

## 2 METHOD

### 2.1 Design

A three‐round electronic modified Delphi (eDelphi) study was used to gain consensus across all participants. A Delphi study refers to a process where participants are asked about an issue and the results of each round are reported to participants before the next round commences until a consensus is met by all participants.^33^

The number of rounds was set to a maximum of three due to project constraints; this is in line with other Delphi studies, which typically range from three to five rounds.^34^, ^35^ Consensus was set at 75% or more of participants in agreement, following guidelines suggesting consensus figures between 70% and 80%, while also utilising multiple rounds and brief thematic analysis of comments provided.^33^, ^36^

A Delphi method was used as it provided an anonymous platform where participants ranked items on level of importance and could give a clear direction on the psychological theme to prioritise in a future intervention study and ways of delivering the intervention. The purpose of this study was to agree on a starting point from which to co‐develop an intervention; therefore, at this stage, it was not necessary to gather detailed information on people's experiences and preferences. The Delphi method allows relatively fast consensus to be generated across larger numbers of people (compared with other methods).

### 2.2 Patient and public involvement (PPI)

Two young people with an LTC provided feedback on the written protocol that included the overall aim and procedures of the project. Additionally, we consulted with another six young people with LTCs on an early draft of the study questionnaire (the four questions). Subsequently, we revised the wording of the questionnaire to improve clarity in line with feedback. Once the project began, a PPI group consisting of two young people with LTCs and one parent of a young person with an LTC met online via a discussion forum with the research team to give feedback on the research process, the lay report of the results and the dissemination plan. Changes were made to the lay report to increase clarity and appeal, and the dissemination plan was altered to be more inclusive for both younger and older children and their families.

### 2.3 Participants

#### 2.3.1 Recruitment

Individuals meeting the following eligibility criteria were included in the study:

1. Young people aged 10–18 years old with one or more of the LTCs asthma, diabetes and epilepsy.
2. Parents or guardians of young people aged 10–18 years old with one or more of the LTCs asthma, diabetes and epilepsy.
3. Professionals working with young people aged 10–18 years old with LTCs from the following fields: healthcare professionals (HCPs) (such as nurses, doctors and clinical psychologists), teachers and social workers.

Young people and parents/guardians were recruited via a newsletter, an online advert and an online public advisory board of eNurture, potentially reaching hundreds of young people and their families. To increase participation, we also used online adverts in school newsletters for two secondary schools in the Midlands, UK. These schools were independent (fee paying) and were part of the same network of schools. Professionals were recruited through workplaces, including the secondary schools above (e.g., for school nurses and teachers), and existing contacts from within the research team, using snowball sampling.

#### 2.3.2 Ethical considerations

The study gained full ethical approval from City University of London Health Services Research and Management Proportionate Review Committee (Reference: ETH2122‐0279). All participants were asked to consent before taking part in the study; however, those aged under 16 were also asked to gain consent from a parent/guardian. A paediatric clinical psychologist on the research team was available for consultation if any participant declared that they were feeling distressed during the study. All members of the research team had current Disclosure and Barring Service certificates to allow them to work with children.

### 2.4 Materials

The eDelphi questionnaire consisted of four questions designed to assess participant preferences for the focus, tools and content of a future intervention to improve mental wellbeing (see Appendix A for the round 1 questions). The online survey software, Qualtrics (https://qualtrics.com), was used to host the questionnaire.

#### 2.4.1 Question 1 (Focus of key mental health themes to be addressed)

Participants were asked about psychological or emotional themes that young people with a long‐term health condition may experience (e.g., anxiety, depression, social isolation). The question consisted of a list of emotional/psychological themes, which participants were asked to rank in order of importance from most important to least important by clicking and dragging items in the list with the most important at the top (see Appendix A for exact question wording).

The items for the initial list in round 1 (in Appendix A) were identified following a meta‐review of systematic reviews of common psychological challenges experienced by children with asthma, diabetes and epilepsy conducted by the authors. The items in the list were presented in a random order. For the first round, there were also free text boxes where participants could write comments about the items in the list, explain their choice of ranking further and suggest any additional items (not already in the list) that they thought should be included.

#### 2.4.2 Question 2 (Tools: Delivery of content)

Question 2 asked about whether an intervention to improve emotional wellbeing would be better delivered as one‐to‐one support (with a therapist or HCP), in a group or a combination of both. In this question (in Appendix A), participants could only select one answer rather than ranking their preferences. The three options are the most common modes of delivery and were chosen using the expertise of the multidisciplinary research team, which included: (1) a team of academic researchers with expertise in (i) mental wellbeing in people with LTCs, (ii) interventions to improve mental wellbeing and (iii) human–computer interactions; (2) a consultant paediatrician and academic researcher; (3) a senior paediatric nurse and (4) a consultant paediatric clinical psychologist.

#### 2.4.3 Question 3 (Tools: Preferred digital technology)

Question 3 looked at potential digital technologies that could be used to deliver an intervention (e.g., a website, an app, a smartwatch, etc.). The items (see Appendix A) were drawn from relevant systematic reviews described in the introduction^29^, ^30^, ^31^ and presented in a random order. For the first round, there were also free text boxes for comments on the list items, explaining ranking decisions and for additional items.

#### 2.4.4 Question 4 (Content)

Question 4 asked about preferences for different types of emotional support that might form the basis of a future intervention (e.g., CBT, mindfulness, general counselling). The list of items to rank (see Appendix A) was created using information on existing interventions^29^, ^30^, ^31^ and using the expertise of the multidisciplinary research team (detailed in Question 2). Some items were included that might be difficult to deliver digitally, for example, family therapy. This was partly because we did not want to exclude anything due to difficulty at this stage, and because there is evidence that delivering techniques such as family therapy digitally is possible and has shown improvement in adolescent mental health.^37^ The items were presented in a random order. Again, there were free text boxes for comments on the list items and for additional items, for the first round.

Demographic information collected at round 1 included:

1. Participant type (whether the participant was a young person, parent/guardian or HCP).
2. Information about the young people, including age, gender identity, LTC/s and approximate duration since diagnosis. This information was reported by the young person themselves or by parents/guardians about their child or children. We did not collect this information about the parents/guardians themselves.
3. Information about the HCP' specialty (e.g., asthma, diabetes, epilepsy care or other specialty), job role/s (e.g., nurse, doctor, psychologist) and length of time spent in the role/s.

### 2.5 Procedure

This project followed an iterative process aiming to gain consensus and was completed over 2 months, between 21 April 2022 and 21 June 2022.

In round 1, participants were sent a link to the eDelphi questionnaire via email with instructions to complete the questions within 2 weeks and to contact the research team with any problems or questions. Participants were sent reminders after the first week if they had not completed the questionnaire.

Within 1 week after the first round had closed, the data were analysed and a modified version of the round 2 questionnaire was created. A brief summary of the results was sent to the participants as part of the round 2 questionnaire, which again was open for 2 weeks with a reminder sent halfway. Round 3 (the final round) followed the same procedure, after which the young people and parents were offered vouchers for taking part.

### 2.6 Data analysis

As part of the Delphi study process, results from each round were disseminated and presented to participants as part of the following round. The question set was revised based on analysis of the previous findings; for example, removing items with low ranking, rewording questions that caused confusion and removing questions with a consensus.^38^, ^39^

Data from the first round were analysed by calculating the percentage of participants who had rated each item as first, second, third, fourth and so forth, for questions that involved ranking items by importance. To select the items for the second round, we kept items from round 1 where at least 10% of participants ranked them as either first, second or third most important and we removed those not meeting the criteria. New items were added following brief thematic analysis of suggestions from participants within the free text boxes.^40^ Any questions meeting consensus were removed for the next round—this meant at least 75% of participants had selected an item as the most important or useful (ranked first in the list).

A similar analysis was carried out on the round 2 data. However, to create lists of items to rank for the third round, the criteria were stricter: we only kept the items that more than 10% of participants had ranked as most important (first in the list) and removed the rest. There were no further (new) items added. Any questions meeting consensus were removed for the next round.

The final round 3 data were analysed by calculating percentages of participants selecting items as first, second, third and so forth. Where a question had still not reached consensus for this round, the results were simply reported by percentages of participants choosing each item as most important. A series of post hoc chi‐squared analyses were also conducted to see if percentages of participants choosing each item as most important differed by LTC and participant type.

## 3 RESULTS

### 3.1 Participant's characteristics

Fifty‐nine participants took part in the study: 26 young people with asthma, diabetes and/or epilepsy; 23 parents/guardians of young people with the specified LTCs and 10 health professionals working with young people with LTCs. The age range of the young people was 11–18 years old, with a mean age of 14.5 (SD 2.12) and mode of 15 years old as reported by the young people themselves or by the parents/guardians about their child (see Table 1 for detailed demographic information). Most of the young people identified as female (74%), with 24% male and 2% identifying as nonbinary. Asthma was the most frequent LTC (58%), followed by epilepsy (21%) and diabetes (15%). Six per cent of young people had other LTC/s in addition to one of the three main LTCs. Over half of the participants reported having the LTC/s for their whole life or for at least 10 years (29% and 24%, respectively).

**Table 1:** Demographic information of YP (self‐reported, reported by parents/guardians and total).

	YP self‐report (*n* = 26)		Parental report of YP (*n* = 23)		Total (YP plus parental report, *n* = 49)	
	*n*	%	*n*	%	*n*	%
Age of young person						
11 years old	2	8	2	9	4	8
12 years old	2	8	5	22	7	14
13 years old	2	8	2	9	4	8
14 years old	2	8	1	4	3	6
15 years old	7	27	7	30	14	29
16 years old	3	12	3	13	6	12
17 years old	4	15	2	9	6	12
18 years old	4	15	1	4	5	10
Total	26	100	23	100	49	100
Gender identity of young person						
Female	20	77	16	70	36	74
Male	6	23	6	26	12	24
Nonbinary	0	0	1	4	1	2
Total	26	100	23	100	49	100
Long‐term condition (LTC)^a^						
Asthma	15	56	16	62	31	58
Diabetes	6	22	2	8	8	15
Epilepsy	5	19	6	23	11	21
Other LTC	1	4	2	8	3	6
Total	27	100	26	100	53	100
Duration of LTC						
<1 year	1	4	1	4	2	4
1–2 years	2	8	1	4	3	6
3–5 years	5	19	4	17	9	18
6–10 years	5	19	4	17	9	18
>10 years	5	19	7	30	12	24
My whole life	8	31	6	26	14	29
Total	26	100	23	100	49	100

Abbreviations: LTC, long‐term condition; YP, young people.

^a^ Participants may select more than one LTC.

Table 2 shows information about the HCPs who participated in the study. Most of the HCPs were paediatric nurses (64%), with the remaining participants being either paediatricians, paediatric psychologists or having other roles. Diabetes was the most common specialism (38%), and half of the participants had been in their role for more than 10 years.

**Table 2:** Job role information of healthcare professionals.

	*n*	%
Job role^a^		
Paediatrician	1	9
Paediatric nurse	7	64
Paediatric psychologist	1	9
Other	2	18
Total	11	100
Specialism^a^		
Asthma	2	15
Diabetes	5	38
Epilepsy	2	15
Other specialisation	2	15
No specialisation or not relevant	2	15
Total	13	100
Length of time in role		
Less than 1 year	1	10
1–2 years	1	10
3–5 years	2	20
6–10 years	1	10
More than 10 years	5	50
Total	10	100

^a^ Participants may select more than one job role and specialism.

Most young people and parents were recruited from two schools in the Midlands, England, and most of the HCPs were working in London NHS Trusts. It was not possible to calculate the exact percentages of participants recruited from each source due to the questionnaire answers being anonymous. This information was deduced from participants expressing interest during recruitment following adverts sent at different times.

### 3.2 Participant retention

Fifty‐nine participants took part in round 1 and 54 participants took part in rounds 2 and 3. Three young people, one parent and one HCP dropped out in rounds 2 and 3 (although this may not necessarily be the same participants dropping out in each round). The retention rate was therefore 92% by the final round.

### 3.3 Focus of key mental health themes to be addressed (Question 1)

The psychological themes that were prioritised in each round are presented in Tables 3a (round 1), 3b (round 2), and 3c (round 3). Only the items meeting above the 10% cutoff for participants rating them as either first, second or third most important in round 1 (Table 3a) were put forward to round 2 (see Table 3b). A few additional items were added to round 2 (see Table 3b), including dealing with unsupportive adults and a lack of control over symptoms. Dealing with unsupportive adults was kept in for round 2 because although it did not meet the cutoff criteria, there were several references to this in the qualitative comments. This item was therefore reworded to reflect participants' experiences. The stricter >10% cutoff for participants rating items first in round 2 resulted in four themes to be ranked in round 3 (see Table 3c).

**Table 3a:** Psychological/emotional themes (Question 1) ordered by percentage of participants choosing item as most important (first): Round 1.

Psychological/emotional theme	Percentage of participants rating item as most important	Percentage of participants rating item as second most important	Percentage of participants rating item as third most important
Anxiety (feeling worried or afraid on most days of the week)	26.32	26.32	17.54
Depression (feeling very sad on most days of the week)	15.79	10.53	17.54
Wanting to appear ‘normal’ and the same as your friends/peers	15.79	12.28	12.28
Denial of having the condition (believing you do not have the condition when you have been told by health professionals that you have it)	10.53	8.77	8.77
Lack of confidence in yourself	8.77	10.53	12.28
Worry about the future (e.g., the possibility of future health issues for yourself)	7.02	5.26	12.28
Poor sleep	7.02	7.02	5.26
Dealing with unsupportive adults (grown‐ups who do not understand)	5.26	1.75	5.26
Fear of possible medication side effects (side effects are consequences of the medication that are not always nice)	3.51	8.77	1.75
A fear of social situations	0.00	8.77	7.02

**Table 3b:** Psychological/emotional themes (Question 1) ordered by percentage of participants choosing item as most important (first): Round 2.

Psychological/emotional theme	Percentage of participants rating item as most important	Percentage of participants rating item as second most important	Percentage of participants rating item as third most important
Dealing with unsupportive adults (e.g., teachers who do not understand or believe my symptoms)^a^	28.30	11.32	5.66
Depression (feeling very sad on most days of the week)	18.87	5.66	15.09
Anxiety (feeling worried or afraid on most days of the week)	13.21	33.96	7.55
Wanting to appear ‘normal’ and the same as your friends/peers	11.32	7.55	5.66
Worry about the future (e.g., the possibility of future health issues for yourself)	7.55	7.55	15.09
Lack of control over symptoms or being unable to predict symptoms	7.55	5.66	13.21
Issues to do with exercise/sports, for example, worrying about symptoms getting worse with exercise	5.66	7.55	3.77
Denial of having the condition (believing you do not have the condition when you have been told by health professionals that you have it)	3.77	1.89	3.77
Stress	1.89	3.77	15.09
Anger	1.89	0.00	1.89
Lack of confidence in yourself	0.00	7.55	11.32
Issues to do with eating, for example, overeating or keeping to a healthy diet	0.00	7.55	1.89

^a^ Added to round 2 with modified wording (despite not meeting round 1 criteria) following thematic analysis of suggestions for additional items.

In round one (Table 3a), the psychological themes that were considered most important were anxiety (ranked first by 26% of participants), depression (16%) and wanting to appear ‘normal’ and the same as your friends/peers (16%). In the second round (Table 3b), 28% of the participants thought dealing with unsupportive adults was most important, 19% thought depression was most important and 13% of participants thought anxiety was most important. The third and final round of the eDelphi (see Table 3c) showed that 44% of the participants reported anxiety as their emotional theme priority, 38% reported wanting to appear ‘normal’ as their priority, followed by depression (9%) and dealing with unsupportive adults (8%). Consensus was not met in this question, but anxiety and wanting to appear ‘normal’ were reported as most important by the majority of participants.

**Table 3c:** Psychological/emotional themes (Question 1) ordered by percentage of participants choosing item as most important (first): Round 3.

Psychological/emotional theme	Percentage of participants rating item as most important	Percentage of participants rating item as second most important	Percentage of participants rating item as third most important
Anxiety (feeling worried or afraid on most days of the week)	44.00	36.00	18.00
Wanting to appear ‘normal’ and the same as your friends/peers	38.46	25.00	19.23
Depression (feeling very sad on most days of the week)	8.51	21.28	36.17
Dealing with unsupportive adults (e.g., teachers who do not understand or believe my symptoms)	7.69	17.31	26.92

### 3.4 Tools to use for psychological interventions (Questions 2 and 3)

#### 3.4.1 Question 2 (Tools: Delivery of content)

In terms of the best way to help young people with LTCs improve their emotional wellbeing, participants had three options: group support, one‐to‐one and a mix of one‐to‐one and group support. For this question, we reached consensus in the first round with 75% of the participants preferring a mix of one‐to‐one and group support, 15% preferring just group support and 10% preferring one‐to‐one support.

#### 3.4.2 Question 3 (Tools: Digital technology)

Regarding technology to deliver the intervention, Tables 4a, 4b, 4c show the changes in the question items from round 1 to round 3 along with percentages of participants ranking each item as a priority. All the items in round 1 met the cutoff criteria (see Table 4a) and were therefore included in round 2. There were no new items to add to round 2 following the qualitative analysis of comments. Only three items in round 2 met the stricter cutoff (see Table 4b) and were included in round 3. A mobile app was consistently ranked first and almost reached consensus with 73% of participants ranking it as the top technology to use by round 3. Something that measures your body was second most preferable (19%) and virtual reality was third (8%) in round 3 (Table 4c).

**Table 4a:** Digital technology preferences (Question 3) ordered by percentage of participants choosing item as most important or useful (first): Round 1.

Technology	Percentage of participants rating item as most important	Percentage of participants rating item as second most important	Percentage of participants rating item as third most important
An app on your phone	61.40	21.05	10.53
Something that measures your body (e.g., your heartbeat) and can send you a message. For example, a smartwatch that sends you a message with some relaxation exercises if your heart is beating fast	14.04	21.05	17.54
A website for information	8.77	31.58	14.04
VR (virtual reality) headsets (e.g., used as a distraction to reduce pain)	7.02	5.26	14.04
Video games	7.02	3.51	14.04
Emails or websites with links to videos, quizzes, games and other online activities	1.75	17.54	29.82

**Table 4b:** Digital technology preferences (Question 3) ordered by percentage of participants choosing item as most important or useful (first): Round 2.

Technology	Percentage of participants rating item as most important	Percentage of participants rating item as second most important	Percentage of participants rating item as third most important
An app on your phone	62.26	22.64	7.55
VR (virtual reality) headsets (e.g., used as a distraction to reduce pain)	15.09	13.21	13.21
Something that measures your body (e.g., your heartbeat) and can send you a message. For example, a smartwatch that sends you a message with some relaxation exercises if your heart is beating fast	15.09	35.85	13.21
Video games	3.77	9.43	18.87
A website for information	3.77	11.32	32.08
Emails or websites with links to videos, quizzes, games and other online activities	0.00	7.55	15.09

**Table 4c:** Digital technology preferences (Question 3) ordered by percentage of participants choosing item as most important or useful (first): Round 3.

Technology	Percentage of participants rating item as most important	Percentage of participants rating item as second most important	Percentage of participants rating item as third most important
An app on your phone	73.08	21.25	5.77
Something that measures your body (e.g., your heartbeat) and can send you a message. For example, a smartwatch that sends you a message with some relaxation exercises if your heart is beating fast	18.52	57.41	24.07
VR (virtual reality) headsets (e.g., used as a distraction to reduce pain)	7.55	20.75	71.70

### 3.5 Content of psychological interventions (Question 4)

In terms of wellbeing support, the highest rated item in this first round was a ‘combination of any of these’ with 50% ranking this option first (see Table 5a). This preference for a mixture of therapies will be noted for future intervention development. However, it was decided to remove this item going into round 2, to help gather more detailed information about which therapies should be combined. All the other items in round 1 met the criteria to be included in round 2.

**Table 5a:** Psychological intervention preferences (Question 4) ordered by percentage of participants choosing item as most important or useful (first): Round 1.

	Percentage of participants rating item as most important	Percentage of participants rating item as second most important	Percentage of participants rating item as third most important
Combination of any of these	50.00	3.57	5.36
Changing unhelpful thoughts and behaviours into more helpful ones (cognitive behavioural therapy)	17.86	21.43	16.07
Things to change behaviours, such as diet or sleep (behavioural therapies)	12.50	28.57	19.64
Relaxation and focusing on the present moment (mindfulness)	7.14	17.86	21.43
General counselling (talking to a trained counsellor or therapist about issues)	5.36	7.14	10.71
Working out what is important and how to do more of it (acceptance and commitment therapy)	3.57	14.29	14.29
Supporting all the family (family therapy)	3.57	7.14	12.50

Another amendment for round 2 was to split ‘relaxation and focusing on the present moment (mindfulness)’ into ‘relaxation and deep breathing exercises’ and ‘focusing on the present moment (mindfulness)’ (Table 5b). This was to reflect qualitative (free text) comments from a few participants who had suggested ‘breathing exercises’ as an additional form of wellbeing support. An extra two options were also added for round 2 based on free text responses in round 1 (Table 5b). There were therefore nine options to be ranked in round 2 (Table 5b).

**Table 5b:** Psychological intervention preferences (Question 4) ordered by percentage of participants choosing item as most important or useful (first): Round 2.

	Percentage of participants rating item as most important	Percentage of participants rating item as second most important	Percentage of participants rating item as third most important
Education or training for school staff and students about long‐term conditions	36.54	17.31	9.26
General counselling (talking to a trained counsellor or therapist about issues)	23.08	25.00	9.63
Support from peers or friends	13.46	13.46	5.77
Changing unhelpful thoughts and behaviours into more helpful ones (cognitive behavioural therapy)	9.62	15.38	1.92
Working out what is important and how to do more of it (acceptance and commitment therapy)	5.77	1.92	7.69
Supporting all the family (family therapy)	5.77	15.38	17.31
Focusing on the present moment (mindfulness)	3.85	3.85	23.08
Relaxation and deep breathing exercises	1.92	3.85	9.62
Things to change behaviours, such as diet or sleep (behavioural therapies)	0.00	3.85	15.38

‘Education or training for school staff and students about LTCs’ was rated highest in round 2, with 37% of participants placing it first. Three items were ranked first by over 10% of participants and therefore were included in round 3 (Table 5c). In the final round, ‘general counselling’ was the highest rated, with 54% ranking it top, education or training for school staff and students was ranked second (28%) and support from peers and friends was ranked third (19%).

**Table 5c:** Psychological intervention preferences (Question 4) ordered by percentage of participants choosing item as most important or useful (first): Round 3.

	Percentage of participants rating item as most important	Percentage of participants rating item as second most important	Percentage of participants rating item as third most important
General counselling (talking to a trained counsellor or therapist about issues)	53.85	17.31	28.85
Education or training for school staff and students about long‐term conditions	27.78	46.30	25.93
Support from peers or friends	18.87	35.85	45.28

### 3.6 Participant group differences

Exploratory post hoc *χ* ^2^ analyses or Fisher's exact tests were conducted to see if there were any differences in the final round results by LTC (asthma, *n* = 30; diabetes, *n* = 11; epilepsy, *n* = 10; excluding three HCPs with no specialism) and by participant type (young person, *n* = 23; parent, *n* = 22; HCP, *n* = 9), using a Bonferroni corrected *α* of .025. Comparisons of the percentage of participants ranking each option as first in the final round by LTC showed no significant differences (*p* values ranged from .037 to .0804). Likewise, there were no significant differences by participant type (*p* values ranged from .030 to .878).

## 4 DISCUSSION

The present study investigated the psychological themes and intervention preferences of young people with the three most common LTCs: asthma, epilepsy and diabetes. Anxiety was found to be the most important psychological challenge that young people with an LTC may experience, with 44% ranking it as the most important theme. Participants identified a preference for a combination of one‐to‐one and group support, reaching a 75% consensus in round 1. An app for a mobile phone was ranked as the most useful technology in all three rounds, getting close to consensus with 73% ranking it highest in round 3. General counselling was identified as the best form of emotional wellbeing support for young people, with 54% of participants rating it as most important or useful in round 3. Additionally, half the participants expressed a preference for a combination of support types in round 1. The findings suggest that a mobile app intervention addressing anxiety and feelings of wanting to appear ‘normal’ using a combination of tailored personalised elements as well as group interaction elements, would be acceptable for young people.

Adolescents and young adults are frequent users of the Internet and are commonly seen as the ‘digital native generation’ due to their familiarity and ease with digital technology from an early age.^41^ Consequently, digital technologies are increasingly being used to share information and engage young people in their healthcare.^42^, ^43^ Online interventions can reduce symptoms and feelings of depression, distress and social isolation, especially in young populations.^44^, ^45^ In particular, mobile app‐based counselling interventions have been found to be effective for improving mental health.^46^, ^47^, ^48^ Almost all children aged 12–17 use apps or sites for communicating via message or call and for social media^49^; therefore, it is likely that an app will be appealing and accessible to the young population.

The results of the study identified anxiety and the desire to appear ‘normal’ as the highest ranked psychological themes, which may be experienced by young people with LTCs. This implies that psychological interventions targeting anxiety might also need to address related issues of self‐perception and social comparison. It would be interesting to see whether anxious feelings are related to social anxiety, health‐related anxiety or are more generalised. Children with LTCs may have higher levels of social anxiety and less social contact with peers; however, this relationship appears to be attenuated by self‐esteem.^50^ Understanding the potential link between anxiety and desire to appear ‘normal’ can guide the development of more effective and comprehensive interventions that address both aspects simultaneously.

Participants valued an intervention approach that provides a broad range of psychological techniques and that offers both individual and group support. Group therapy has been shown to be as effective as individual therapy for supporting mental health in adults.^51^ However, there is limited evidence to suggest the advantages of attending both group and individual therapy concurrently, except in specific circumstances such as for people with posttraumatic stress or avoidant personality disorder.^52^, ^53^ For young people with LTCs, a peer support group may be more suitable than more formal group therapy (as suggested in the comments boxes and selected as first choice by almost 20% of participants in round 3). Social support from peers and people delivering the intervention plays a key role in supporting adolescents and young people to access an intervention, gain new skills, develop a more hopeful view for the future and may help sustain improvements to mental health beyond the end of the intervention.^54^

Peer‐support initiatives have been successful in decreasing both loneliness and isolation in young people with LTCs. In this context, peers are often defined as ‘nonprofessional’ with experience of, or familiarity with, a condition. Peer support interventions have been associated with increased self‐efficacy and condition management in young people with diabetes^55^ and with improved coping, self‐confidence and decreased loneliness in children with asthma.^56^ Peer support for young people with epilepsy (either in person or online) was also shown to offer a unique type of care which included both emotional and instrumental support.^57^ A recent systematic review of technology‐based peer support interventions for YP with LTCs found that such interventions were feasible and acceptable with positive impacts on social support, but there were not enough RCTs to support firm conclusions on their overall efficacy in other areas such as isolation and symptom management.^58^

Interestingly, the preferred intervention type among participants in round 3 was ‘general counselling’. Considering that the participants favoured a combination of different types of support, individual counselling might be provided alongside other types of support such as education in schools and peer support. In practice, this might be difficult to achieve, especially using an app for delivery. Further research and co‐design with young people with LTCs and following the WHO Youth Centred Digital Health Interventions guidance^59^ will be necessary to develop suitable interventions that are acceptable, practicable and effective.

### 4.1 Limitations

The necessary brief description of some complex ideas (such as psychological issues and therapeutic techniques) reduced the ability to fully explain these terms. As a result, participants may have chosen items that they were more familiar with (e.g., choosing ‘general counselling’ over acceptance and commitment therapy). Therefore, further consultation will be necessary so that more complex ideas can be more fully explained and explored. As noted in the design section, the Delphi method does lack depth; however, it is a relatively fast and anonymous way to gain consensus across a larger number of people. For the current study, we wanted relevant stakeholders to help us identify what psychological themes and what interventions we need to prioritise in future. However, we acknowledge that this method does not allow for a detailed understanding of some of the nuances associated with mental wellbeing difficulties and therapeutic techniques.

The study sample may not be representative of the population. Due to time and resource restrictions, health professionals were recruited via snowball sampling and most parents and young people were recruited from two independent secondary schools in the Midlands. Help‐seeking and decision‐making behaviour in LTCs may be affected by factors such as geographical location and socioeconomic status.^60^ More data could have been collected, including parents' gender, socioeconomic status and place of work for the HCPs, this would have given us a more detailed picture of the participants included in this study. We were careful to only collect data directly linked to our research question, to minimise participants' burden and attrition over time (data were collected at multiple time points). Furthermore, the study was conducted as the United Kingdom was coming out of coronavirus disease restrictions, which may have influenced the results, for example, by exacerbating feelings of anxiety.

Despite these limitations, the study has provided important information about intervention preferences in consultation with a range of participants including young people with LTCs and key people involved in their care (parents and HCPs). The number of participants was also large for a Delphi method study with high participant retention across the three rounds.

## 5 CONCLUSION

Young people with LTCs identified a desire for an app‐based intervention primarily focused on addressing anxiety. It is hoped that such an intervention will be effective given previous efficacy of apps with young populations. Furthermore, a preference for a mixed approach of both group and individual support was highlighted—an approach that may present practical difficulties but could potentially increase the chance of successful outcomes. Finally, participants showed a preference for a mixture of therapeutic approaches, specifically with general counselling being the preferred option provided. Using the Delphi method in research is a useful tool that could be used more often to gather user feedback before creating a bespoke intervention.

## AUTHOR CONTRIBUTIONS

**Jennie Brown**: Writing—review and editing; writing—original draft; formal analysis; project administration; data curation; supervision; methodology; visualisation; investigation. **Lauren Cox**: Writing—original draft; project administration; investigation; methodology. **Kathleen Mulligan**: Writing—original draft; methodology; project administration; conceptualisation. **Stephanie Wilson**: Methodology; conceptualisation; writing—original draft. **Michelle Heys**: Methodology; conceptualisation; writing—original draft; resources. **Polly Livermore**: Conceptualisation; writing—original draft; methodology; resources. **Suzy Gray**: Writing—original draft; conceptualisation; methodology; resources. **Angeliki Bogosian**: Conceptualisation; funding acquisition; investigation; writing—original draft; methodology; writing—review and editing; supervision; resources; formal analysis.

## CONFLICT OF INTEREST STATEMENT

The authors declare no conflict of interest.

## ETHICS STATEMENT

The study gained full ethical approval from City University of London Health Services Research and Management Proportionate Review Committee (Reference: ETH2122‐0279). All participants aged 16 and above gave informed consent to take part. Participants aged 11–15 years also obtained consent from their parents/guardians.

## Data Availability

Data are available via City University of London data repository: https://doi.org/10.5255/UKDA-SN-857049.

## APPENDIX A QUESTIONNAIRE FOR ROUND 1

**Question 1**

Below you will find a list of psychological or emotional themes that young people with a long‐term health condition may experience. Please order them according to how important you think they are by clicking and dragging the phrases up and down the list. The most important should be at the top and the least important should be at the bottom.

**Table:**

Anxiety (feeling worried or afraid on most days of the week)
Depression (feeling very sad on most days of the week)
Denial of having the condition (believing you do not have the condition when you have been told by health professionals that you have it)
Fear of possible medication side effects (side effects are consequences of the medication that are not always nice)
Dealing with unsupportive adults (grown‐ups who don't understand)
Wanting to appear ‘normal’ and the same as your friends/peers
Poor sleep
A fear of social situations
Worry about the future (e.g., the possibility of future health issues for yourself)
Lack of confidence in yourself

Please use the box below to write any comments you have on the list above. For example, why do you think the themes you put at the top of the list are important?

If you have no comments, then please leave this blank.

Please use the box below to tell us if there are any other psychological or emotional themes (not listed above) that young people with a long‐term health condition may experience.

If you have no comments, then please leave this blank.

**Question 2**

Which would be the best way to help young people with long‐term health conditions improve their emotional wellbeing? (Please choose one)

1. Group support with other young people who have a long‐term condition
2. One‐to‐one support (e.g., with a therapist or other health professional)
3. A mix of one‐to‐one and group support

**Question 3**

Below you will find a list of technologies we could use for our future study. Please order them according to how important or useful you think they are by clicking and dragging the phrases up and down the list. The most important should be at the top and the least important should be at the bottom.

**Table:**

A website for information
An app on your phone
Emails or websites with links to videos, quizzes, games and other online activities
VR (virtual reality) headsets (e.g., used as a distraction to reduce pain)
Video games
Something that measures your body (e.g., your heartbeat) and can send you a message. For example, a smartwatch that sends you a message with some relaxation exercises if your heart is beating fast

Please use the box below to write any comments you have on the list of technologies above. For example, why do you think the technologies you put at the top of the list are important?

If you have no comments, then please leave this blank.

Please use the box below to tell us if there are any other technologies (not listed above) that young people with a long‐term health condition may experience or want to use.

If you have no comments, then please leave this blank.

**Question 4**

Below you will find a list of emotional wellbeing support we could develop in the future. Please order them according to how important or useful you think they are by clicking and dragging the phrases up and down the list. The most important should be at the top and the least important should be at the bottom.

**Table:**

Changing unhelpful thoughts and behaviours into more helpful ones (cognitive behavioural therapy)
Things to change behaviours, such as diet or sleep (behavioural therapies)
Relaxation and focusing on the present moment (mindfulness)
Working out what is important and how to do more of it (acceptance and commitment therapy)
Supporting all the family (family therapy)
General counselling (talking to a trained counsellor or therapist about issues)
Combination of any of these

Please use the box below to write any comments you have on the list of support above. For example, why do you think the types of support you put at the top of the list are important?

If you have no comments, then please leave this blank.

Please use the box below to tell us if there are any other types of support (not listed above) that young people with a long‐term health condition may experience or want to access.

If you have no comments, then please leave this blank.
